# A Case of Hepatitis E in Metropolitan New York

**DOI:** 10.7759/cureus.21661

**Published:** 2022-01-27

**Authors:** Sai Harika Pujari, Aditya Chauhan, Surbhi Singh, Mossammat Mansur

**Affiliations:** 1 Department of Internal Medicine, The Brooklyn Hospital Center, Brooklyn, USA; 2 Department of Infectious Diseases, The Brooklyn Hospital Center, Brooklyn, USA

**Keywords:** antivirals, liver failure, enteric transmission, unexplained fever, hepatitis e

## Abstract

A 22-year-old female presented with unexplained, non-specific, constitutional symptoms of fever, chills, rigors, and headaches of more than two-week duration. An extensive workup was done that included labs, imaging, and a liver biopsy. On examination, lymphadenopathy was found, with worsening transaminitis on labs. She was ultimately found to be hepatitis E positive. The disease was self-limited with subsidence of all symptoms with only symptomatic treatment. Hepatitis E virus infection can be a missed diagnosis as it is not common in the United States. An indicated differential diagnosis can save an extensive workup and prevent delayed diagnosis for a patient.

## Introduction

Hepatitis E infection caused by a single-stranded RNA virus is usually self-limited and transmitted by the fecal-oral route causing viral hepatitis. Although the disease is typically self-limited, there have been cases of acute liver failure and extra-hepatic manifestations such as Guillain-Barre syndrome and pancreatitis that must be watched out for.

## Case presentation

A 22-year-old female presented to the hospital with intermittent fever and chills with rigors of two-week duration, self-resolving bilateral eye swelling, and bi-temporal headaches. There was no travel history, no family history of liver disease, sick contacts, or other associated symptoms on review of systems, including gastrointestinal and genitourinary symptoms. Vitals were notable for high-grade fevers up to 102-103 °F. Physical exam was notable for mildly tender posterior cervical lymphadenopathy. Labs were notable for leukopenia, elevated inflammatory markers, and elevated transaminases. Differential diagnoses included but were not limited to infectious, connective tissue disorders, malignancy, and endocrine pathology. Extensive workup including routine cell counts, peripheral smears, urine and blood cultures, HIV antigen/antibody, rapid plasma reagin, toxoplasma, Bartonella, quantiferon, Epstein-Barr virus, cytomegalovirus, herpes simplex virus, alpha 1 antitrypsin, smooth muscle antibody, antinuclear antibody, rheumatoid factor, mitochondrial antibodies, thyroid stimulating hormone, parathyroid hormone, and ceruloplasmin was negative. Hepatitis A, B, and C serologies were negative. However, the patient’s transaminases continued to worsen and up-trended with aspartate aminotransferase as high as 717 U/L and alanine aminotransferase level of 879 U/L with worsening lactate dehydrogenase at 1192 U/L. Radio-imaging included a CT scan of the neck, chest, abdomen, and pelvis, which was essentially negative.

The patient clinically lacked improvement despite empiric broad-coverage antibiotics and continued to have high-grade fevers with other constitutional symptoms such as fatigue and malaise. A liver biopsy was performed in the light of worsening transaminitis, which revealed histopathological features of mild hepatitis. Trichrome stain, periodic acid Schiff-D, iron, and reticulin stains showed no significant pathology. Eventually, the patient’s hepatitis E immunoglobulin M (IgM) tested positive, which provided a reasonable justification for her symptoms. With time and appropriate supportive management, the patient’s transaminases started improving and essentially normalized. The patient was discharged home with outpatient follow-up. The patient remains symptom-free with consistently normal liver function tests after one month of resolution of hepatitis E.

## Discussion

Hepatitis E virus (HEV), as long known, is a single-stranded RNA virus, which is usually self-limited, transmitted via fecal-oral route, and causes viral hepatitis. Its transmission is high among less resourceful countries. The consumption of contaminated food and water is the primary way of transmission, while blood transmission and vertical transmission have also been observed [1]. The genotype common in the United States is 3 and rarely 4, where the transmission is due to undercooked meat consumption, as seen in areas of Europe and East Asia [2,3]. However, African and Asian countries get affected by genotypes 1 and 2, typically sporadic and transmitted by contaminated drinking water. As mentioned above, the infection is usually self-limited and presents with constitutional symptoms such as fever, nausea, and anorexia, but it can also result in vomiting, diarrhea, and hepatomegaly. Rash and pruritus have also been documented in the literature as part of clinical symptomatology. Acute hepatic failure can occur in patients with pre-existing liver disorders or immunocompromised, pregnant, and malnourished individuals.

Various case reports, studies, and series have described HEV infection, including rare cases where patients require liver transplants due to acute liver failure [4,5]. The diagnosis is typically through IgM antibodies, as detected in our patient. If the chronic infection is suspected in a patient, which may be seen in patients with HIV, transplant, and hematological malignancies, an RNA test needs to be obtained and is defined as the presence of RNA for more than six months in either serum or stool [6]. As the disease is self-limited, management is usually supportive. However, physicians need to watch out for extra-hepatic manifestations such as pancreatitis, Guillain-Barre syndrome, poly-radiculopathy, cranial nerve palsies, or seizures [7-9]. However, in patients with immunosuppression, the use of antiviral therapy and reduction of immunosuppression should be considered under the guidance of specialists. Ribavarin is commonly used [10]. Education about the disease and hygiene and sanitation appears crucial in preventing the disease. Unfortunately, the vaccine is not widely available or in use.

The important thing to note from this case report is that although HEV is not as prevalent or as severe as the other hepatitis serologies we commonly test for, including hepatitis A, B, and C, hepatitis E should be suspected in patients with unexplained fever, elevated transaminases, and unclear history. Transaminases can peak up to very high levels, up to 1000s, and confused with a drug-induced liver injury. This case report gives us an example of how an indicated differential diagnosis can sometimes save an extensive workup and prevent a delay in diagnosis for a patient.

## Conclusions

Acute hepatitis E infection must be suspected even in patients with unexplained constitutional symptoms and transaminitis. In this case, waste of resources was seen as invasive tests were performed due to late suspicion that could have been prevented. Timely detection can help manage life-threatening complications in such cases and simple differential diagnosis can save any further delay.
